# Genome analysis of *Plectus murrayi*, a nematode from continental Antarctica

**DOI:** 10.1093/g3journal/jkaa045

**Published:** 2020-12-22

**Authors:** Xia Xue, Anton Suvorov, Stanley Fujimoto, Adler R Dilman, Byron J Adams

**Affiliations:** 1 Precision Medicine Center, Academy of Medical Sciences, Zhengzhou University, Zhengzhou 450000, China; 2 Department of Biology, Evolutionary Ecology Laboratories, and Monte L. Bean Museum, Brigham Young University, Provo, UT, USA; 3 Department of Computer Science, Brigham Young University, Provo, UT, USA; 4 Department of Nematology, University of California, Riverside, CA, USA

**Keywords:** gene loss, genome architecture, genome assembly, genome decay, Plectus murrayi

## Abstract

*Plectus murrayi* is one of the most common and locally abundant invertebrates of continental Antarctic ecosystems. Because it is readily cultured on artificial medium in the laboratory and highly tolerant to an extremely harsh environment, *P. murrayi* is emerging as a model organism for understanding the evolutionary origin and maintenance of adaptive responses to multiple environmental stressors, including freezing and desiccation. The de novo assembled genome of *P. murrayi* contains 225.741 million base pairs and a total of 14,689 predicted genes. Compared to *Caenorhabditis elegans*, the architectural components of *P. murrayi* are characterized by a lower number of protein-coding genes, fewer transposable elements, but more exons, than closely related taxa from less harsh environments. We compared the transcriptomes of lab-reared *P. murrayi* with wild-caught *P. murrayi* and found genes involved in growth and cellular processing were up-regulated in lab-cultured *P. murrayi*, while a few genes associated with cellular metabolism and freeze tolerance were expressed at relatively lower levels. Preliminary comparative genomic and transcriptomic analyses suggest that the observed constraints on *P. murrayi* genome architecture and functional gene expression, including genome decay and intron retention, may be an adaptive response to persisting in a biotically simplified, yet consistently physically harsh environment.

## Introduction

As the top metazoan of Antarctic terrestrial food webs (Wall and Virginia 1999), nematodes contribute to a wide range of soil processes and play a crucial role in soil nutrient cycling (Barrett *et al.* 2008). *Plectus murrayi* is a free-living soil nematode of continental Antarctica, where it has persisted in one of the harshest terrestrial environments on Earth for millions of years (Convey *et al.* 2008). Extremely low temperatures, limited nutrient supply, high osmotic gradients, low water availability, and infrequent periods of time each year when liquid water is available to carry out basic metabolic functions provides a backdrop of multiple environmental stresses to which it must respond and adapt (Fountain *et al.* 1999; Doran *et al.* 2002; John 2007; Fountain *et al.* 2014). *Plectus murrayi* inhabits a wide range of soil environments, reflecting a high dispersal capacity and broad ecological amplitude (Adams *et al.* 2014b; Velasco-Castrillón and Stevens 2014). Endemic to continental Antarctica, most *P. murrayi* are found in soils and limno-terrestrial environments where they have likely persisted from the mid Miocene until the present (Convey 2008; Pugh and Covey 2008; Convey 2009). Given that austral summer temperatures provide only a few days above freezing each year, most individuals must survive multiple years of exposure to intracellular freezing, thawing, and desiccation cycles in order to carry out their entire life cycle. In response to the extremely harsh environmental stressors, the survival strategies of *P. murrayi* have evolved to facilitate anhydrobiosis (Adhikari *et al.* 2009; Adhikari and Adams 2011) and freezing tolerance (Adhikari *et al.* 2010b) at the genomic level. Thus, assembling a complete genome of *P. murrayi* is a valuable first step toward identifying and understanding functional genes that are associated with environmental stress tolerance (Wharton 2002) and even ecological and evolutionary stoichiometry (Elser *et al.* 2000). Moreover, it provides a foundation for a better understanding of patterns of genome evolution and architecture (Nevo 2001), and resolving important nodes in the nematode tree of life (Blaxter and Koutsovoulos 2014). Unlike many organisms from extreme environments, *P. murrayi* can be cultured on monoxenic lawns of *Escherichia coli* OP-50 under laboratory conditions (Adhikari *et al.* 2010a) where, its egg-to-egg lifecycle takes approximately 53–57 days at 15°C (de Tomasel *et al.* 2013). This process yields sufficient numbers of individuals in a relatively short time for genome sequencing and decreases the diversity of complications introduced by contaminant organisms present in environmental samples.

Genome decay (gene loss and genome reduction) is a phenomenon known to occur widely in prokaryotes (Judelson *et al.* 2012; Mandadi and Scholthof 2015; Couce *et al.* 2017) and eukaryotes, perhaps the best examples of this being the loss of significant amounts of genomic and morphological complexity by Tardigrada (Smith *et al.* 2016) and Dicyemids (Kobayashi *et al.* 1999). However, similar examples can be also found throughout Nematoda (Mitreva *et al.* 2005). Such reductions are thought to arise from selection pressures associated with deletion bias (Mira *et al.* 2001), simplification (Smith *et al.* 2016), or increasing specialization (Smith *et al.* 2006; Cramer *et al.* 2011), including parasitism (Olson *et al.* 2012; Wiredu Boakye *et al.* 2017; Lammers *et al.* 2019). We hypothesized that an adaptive response to an increasingly austere environment would entail selection for the maintenance of fewer, but more specialized genes, and strong selection against the maintenance of genes that are advantageous only in more complex ecosystems.

In this study, we sequenced and assembled the genome of *P. murrayi* and compared it with the genome of *C. elegans*. To explore the effect of 5 years of culturing under laboratory conditions, we compared transcriptomes of cultured *P. murrayi* with those extracted directly from frozen field-collected soils. Our primary objective was to make genomic and transcriptomic resources for this emerging model organism publicly available. As part of this effort, our findings shed light on how millions of years of selective pressure by extreme physical drivers, but few biotic influences, have shaped the evolution of a terrestrial metazoan genome.

## Materials and methods

### Specimen collection and maintenance

Populations of *P. murrayi* were collected from Antarctic Dry Valley soils from 2008 to 2009 and maintained in the laboratory on phosphorus sand agar medium at 15°C according to Adhikari et al. (2010a). To test for potential effects of artificial selection due to prolonged laboratory culture, we also isolated populations of *P. murrayi* from field-collected soil samples. For this study, cultures of *P. murrayi* were grown in individual 60 mm petri dishes at 15°C. Culture medium preparations and culture transfers were carried out in a biosafety cabinet under sterile conditions. The final medium consisted of 15 g agar, 965 ml H_2_O, 20 ml BMB (Bold Modified Basal), and 10.33 mg K_2_HPO_4_. The pH was adjusted to 7.0 and then ddH_2_O was added to 1.0 l, and the mixture was autoclaved for 20 min at 120°C. Two grams of sterile sand was poured on cooled plates, which were stored at 4˚C. Thirty microliters of a 1% (w/w) suspension of *E. coli* OP-50 (∼10^9^ cells/ml) were added to each plate using a cotton swab. The OP50-inoculated plates were incubated at 37°C for 2 days. Although the natural environment of *P. murrayi* rarely exceeds 22°C (Doran *et al.* 2002), optimal growth conditions are at 15°C with brief periodic exposure to 26°C (Adhikari et al. 2010a). Therefore, after the 2-day 37°C incubation period, nematodes were added to the plate and incubated at 26°C for 1 week, followed by incubation at 15°C. To keep nematode cultures healthy and fresh, we transferred a piece of agar (containing nematodes) onto the new plates every 3 weeks.

### DNA extraction and library preparation

Populations of *P. murrayi* were washed and rinsed three times with 0.4% hyamine and kept in Ringer’s solution for 15–30 min before DNA extraction. Wizard Genomic DNA Purification Kits (Promega, Madison, WI) were used for extracting DNA from a population of approximately 5,000 nematodes. The genomic DNA was treated with RNaseA to remove any RNAs in the sample. The genomic library was constructed using an Illumina Paired-end DNA sample preparation kit following the manufacturer’s instructions. Because of the difficulties of gathering large amounts of *P. murrayi*, the library was built from a low concentration of total DNA which required amplification. Genomic libraries were sequenced on an Illumina 2000 Genome Analyzer IIx sequencer in paired-end mode with the read length of 76 bp using a single flow cell.

### RNA extraction and library preparation

Soil samples containing wild-caught *P. murrayi* were kept at –20°C, then thawed gradually (–10 °C for 24 h; –4°C for 24 h; 0°C for 24 h; 4°C for 24 h) before individual animals were picked for RNA extraction. Preparation of all RNA sequencing libraries were carried out under identical conditions. Accordingly, wild-caught *P. murrayi* and cultured *P. murrayi* were isolated at room temperature, stored in RNA later solution individually at 4°C and then washed twice with 5% phosphate buffer saline solution (PBS) prior to RNA extraction. Total RNA for transcriptome sequencing was extracted using NucleoSpin RNA kits (Macherey-Nagel). RNA sequencing libraries were prepared with KAPA stranded RNA-seq kits (Roche). QA/QC was carried out using a Bioanalyzer (Agilent) prior to library construction. Single-end sequencing was performed across two lanes on an Illumina HiSeq 2500 in the sequencing center at Brigham Young University. All the resulting fastq files were quality controlled by FastQC (Andrews 2010). Contamination screening was carried out using Diamond (Benjamin Buchfink 2015) and Blobtools (Laetsch and Blaxter 2017).

### Genome assembly

Sequence reads were trimmed in Trimmomatic (V 0.36) (Bolger *et al.* 2014). ILLUMINACLIP: TruSeq3-PE.fa: 2:30:10 was used for removing adapters. Reads with quality lower than 3 were removed by LEADING: 3 and TRAILING: 3, 4-base wide sliding window scanned the reads and cut when the average quality was lower than 15. Reads smaller than 36 bp were dropped. We assembled the whole genome with SOAP de novo (Ruibang Luo 2012) using a 57-mer as calculated by KmerGenie (Chikhi and Medvedev 2014) to be optimal. GapCloser (V 1.12) was used to close the gaps in scaffolds from SOAPdenovo and we ran RepeatMasker (Bergman and Quesneville 2007; Saha *et al.* 2008) to identify the interspersed repeats in the genomic sequences. Benchmarking universal single-copy orthologs (BUSCO) v. 1.1.1 (Simao 2015) was used to evaluate the completeness of our assembled genome by using the metazoa dataset (downloaded in March 2018). Blobtools (Laetsch and Blaxter 2017) was used for contamination screening and low-quality scaffolds and the contaminated sequences were trimmed and excluded. The RNA-seq data are available on NCBI (SRX3784072). The assembled genome was converted to NCBI format and assigned as NCBI bioSample project number SAMN04625768.

### Transcriptome assembly

We used Trinity to assemble the *P. murrayi* raw reads (fastq). The single-end default parameter was used with Bowtie (V 2.2.5) (Langmead and Salzberg 2012). Open reading frames were predicted by Transdecoder (Haas *et al.* 2013). Total annotation was reported from Trinotate (V3.1.1) with outcomes from Trinity (Haas et al. 2013), HMMER (Finn *et al.* 2011), PFAM (Lagesen *et al.* 2007), tmHMM (Krogh *et al.* 2001), GO (Ashburner *et al.* 2000), and RNAMMER (Lagesen *et al.* 2007). Assembled transcriptome metrics showed an acceptable percentage (over 70%) of reads mapping back to each transcriptome indicating qualified assemblies. TransRate scores ranged from 0.1 to 0.59 for *P. murrayi*, which were used for quality assessment, and BUSCO (v. 1.1.1) (Simao 2015) results using the metazoa dataset (downloaded in March 2018) indicated that both transcriptomes have a moderate level of completeness (over 45%). Because we did not utilize a reference genome or transcriptome for our transcriptome assembly, and because some novel transcripts have been detected in our assembly, there may be some partial transcripts contained in both datasets. Additional sequencing from other Antarctic nematodes species is needed to obtain complete transcriptomes.

#### Annotations

*Ab initio* gene-finding was performed by Augustus 2.2.5 (Stanke *et al.* 2008). RNAseq data were taken into account for transcript assemblies generated with PASA47 (Haas *et al.* 2003). Combining the transcriptome assembled by Trinity, EVidenceModeler (EVM) (Haas *et al.* 2008) was used to find consensus annotation based on the data from Augustus, PASA and Trinity for final prediction and annotation in PASA (using the Nematoda database) and Blast2GO (Götz *et al.* 2008).

### Transcriptomes comparison

The transcriptomes assembled byTophat2 (Trapnell *et al.* 2012) and Trinity (Haas *et al.* 2013) with Bowtie2 (V 2.2.5) (Langmead and Salzberg 2012), and the mapping rate of lab-reared and wild-caught *P. murrayi*, both of which were over 90%, are considered moderately complete by BUSCO metrics. The N50 value peak contains over 80% of the expression data, indicative of a robust transcriptome assembly. We used Cufflinks (V 2.2.1) (Trapnell *et al.* 2012) to compare patterns of gene expression between lab-reared and wild-caught *P. murrayi* using *C. elegans* as a reference. Here, we applied TMM normalization to generate a matrix of normalized FPKM values across both datasets. Although the raw fragment counts are used for differential expression analysis, we used the normalized FPKM values to examine expression profiles across different samples, visualized as heatmaps and related expression plots.

### Data availability

The transcriptome data are available at NCBI (PRJNA437987). The Genome sequencing and assembly data are available at NCBI (PRJNA317772) with the accession number (GCA_004785735.1).

Supplementary material is available at figshare DOI: https://doi.org/10.25387/g3.13363646.

## Results

### Genome assembly

In total, 107,770, 211 raw reads were obtained with an average insert size of 550 bp. The original read data comprised 1.93 Gbp with >90% of bases having Phred quality scores greater than or equal to 30 (Q30). The estimated size of the draft genome assembly of *P. murrayi* is 225.71 Mbp with a scaffold N50 of 18,876 bp. The maximum scaffold size is 270,881 bp and GC content is 43.60% (Table 1). Sequencing quality statistics and genome assembly information are shown in Table 1. After trimming the low-quality reads and removing bacterial contaminants (primarily *E. coli* OP-50 from the culture plates), the remaining reads were assembled with an appropriate K-mer (57-mer) as calculated by KmerGenie (Chikhi and Medvedev 2014). The N50 of the assembly is 18,876 bp with approximately 70X coverage. Despite our ability to culture *P. murrayi*, the limitations of harvesting sufficient material for generating high quality sequencing libraries is evidenced by the relatively large number of genome scaffolds that required trimming before performing annotation and gene prediction (146,833 contigs, 117,230 scaffolds). After filtered scaffolds smaller than 10000 bp were removed and we performed repeat contamination screening with nucleotide BLAST (BLASTn; (Altschul 1990)) and BlobTools (Laetsch and Blaxter 2017) to eliminate untargeted sequences, 4505 scaffolds remained for further annotation and prediction. Subsequent gene prediction and annotation is followed from the cleaned draft genome (Table 2). The percentage of complete BUSCOs (Simao 2015) is 77.3%; the single-copy BUSCO frequency is 75.8% (Table 3).

**Table 1:** *Plectus murrayi* draft genome summary

Assembly size (bp)	225,713,687
N50	18,876
N50 length (bp)	84,710,582
GC (%)	43.60
Gene density (gene/Mb)	250.21

**Table 2:** *Plectus murrayi* gene summary

Genes: EVM	
Total number of genes	14,680
Mean gene length (bp)	1,641.809
Exons: EVM	
Number of exons	139,736
Mean number per gene	9.52
Mean length (bp)	257.179
Introns: EVM	
Number of introns	153,487
Mean number per gene	10.46
Mean length (bp)	351.46
UTRs: PASA	
Number of genes having UTR	116,51
Mean UTR length (bp)	64.182
Number of 5ʹUTRs	108,89
Mean 5ʹUTR length (bp)	21.628
Number of 3ʹUTRs	11,365
Mean 3ʹUTRs length (bp)	104.955
Number of mRNA	59,649
Number of transcript	59,649

**Table 3:** The summary of BUSCO

BUSCOs	Percentage (%)	Number
Complete	77.3	756
Complete and single copy	75.8	741
Complete and duplicated	1.5	15
Fragmented	11.1	109
Missing	11.6	113
Total		978

Comparisons of *P. murrayi* and *C. elegans* genomes using resources available through WormBase (https://wormbase.org/) reveal that *P. murrayi* has fewer genes, but a larger genome size, shorter exons, and fewer DNA transposons (Table 4) (Spieth *et al.* 2014). The results from repeatMasker for *P. murrayi* reveal the total number of bases masked in *P. murrayi* is 0.42% compared to 12.59% in *C. elegans*, including tandem repeats and satellite DNA (Table 5).

**Table 4:** Comparison of *P. murrayi* and *C. elegans* genome structure

Genes: EVM	*P. murrayi*	*C. elegans*
Genome size	225.71 million bp	100 million bp
Total number of coding genes	14,680	20,222
Mean gene length (bp)	1.64k	1.83k
Number of exons	139,736	125,702
Mean exons per gene	9.52	5
Mean length (bp)	257.197	1.0k
DNA transposons	6,071 (0.20%)	1,763,494 (1.8%)

### Distribution of alternative splicing modes

The annotation file of *P. murrayi* (gff3 file, Supplementary Table S1) was obtained from PASA. In order to compare *P. murrayi* to *C. elegans* we downloaded their corresponding annotation files from Wormbase(ftp://ftp.wormbase.org/pub/wormbase/releases/WS253/species/c_elegans/PRJNA13758/). We performed an alternative splicing transcriptional landscape analysis on these two species using the AStalavista web server (http://genome.crg.es/astalavista/, 2021). Tables 6 provide the distribution of their alternative splicing modes for coding genes.

**Table 5:** Analysis of repeats in the genome of *P. murrayi*

	Length occupied (bp)	Percentage of Sequence (%)
SINEs	18,955	0.02
LINEs	5,276	0.01
LTR elements	13,245	0.02
DNA transposons	145,952	0.17
Simple repeats	111,341	0.13
Satellites	6,483	0.01
Total interspersed repeats	204,394	0.24

**Table 6:** Alternative splicing model of *P. murrayi*, *C. elegans*

	Alternative	Intron	Alternative	Exon	Other
	acceptor (%)	retention (%)	donor (%)	skipping (%)	events (%)
*Plectus murrayi*	20.54	20.67	15.45	22.94	20.4
*Caenorhabditis elegans*	26.17	14.31	15.80	25.60	18.12

**Figure 1:**
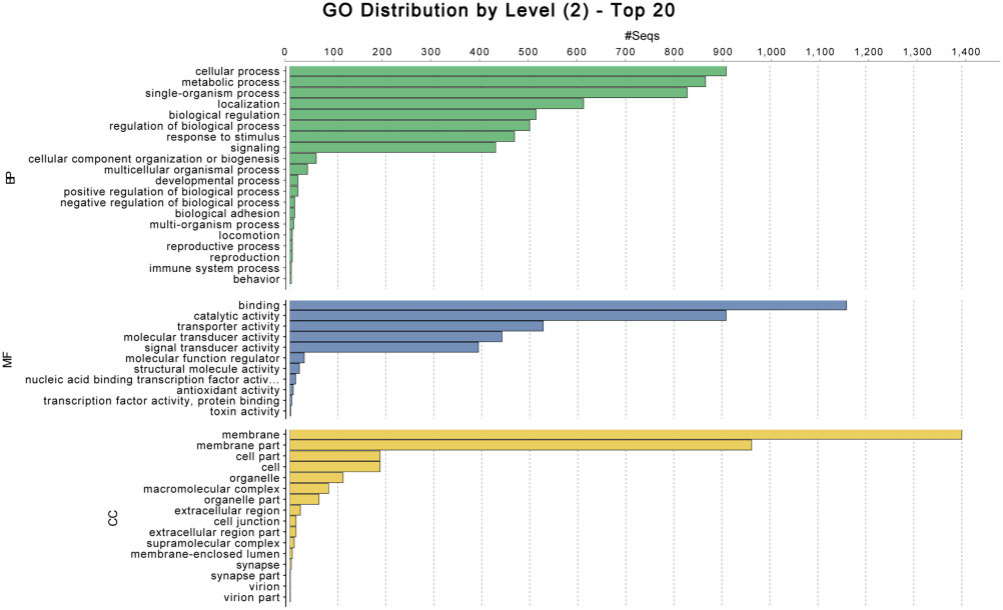
Gene ontology distribution of *P. murrayi* annotation from Blast2GO. BP, biological process; MF, molecular function; CC, cellular component.

**Figure 2:**
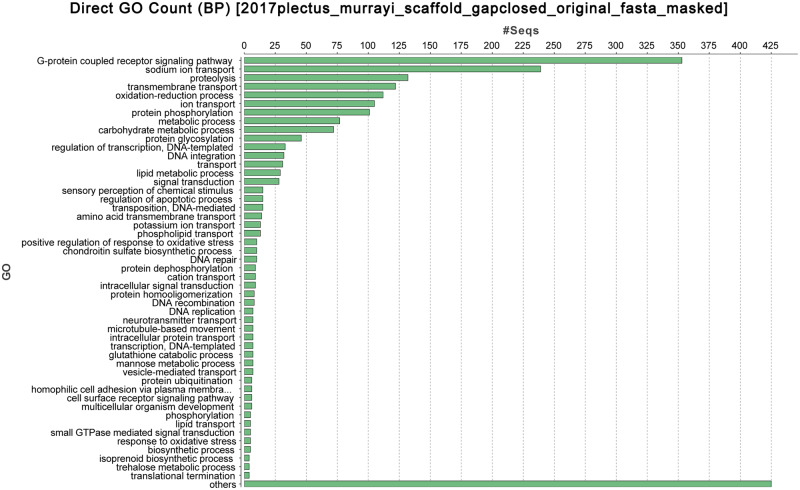
Gene ontology counts of the genome of *P. murrayi*.

The distribution of alternative splicing modes of *P. murrayi* and *C. elegans* shows that intron retention in *P. murrayi* is 20.67% and alternative exon usage is 22.94%, while intron retention in *C. elegans* is 14.31% and alternative exon usage is 25.60%. According to gene prediction and annotation by Augustus (Stanke *et al.* 2008), PASA (Haas *et al.* 2003) and EVM (Haas *et al.* 2008), the total number of *P. murrayi* protein-coding genes is 14,680 (Table 2). The gene ontology (GO) term and pathway analysis by Blast2GO (Götz *et al.* 2008) is presented in Figures 1 and 2.

Compared to *C. elegans*, the genome of *P. murrayi* contains relatively fewer sequences associated with behaviors and locomotion, and more than that it involves metabolism and cellular processing. This observation is consistent with an evolutionary response to increased demand for responding to physical abiotic drivers, as well as decreased demand for gene products involved in biotic interactions, such as complex behaviors, competition and predation (Convey 1996; Hogg *et al.* 2006). However, since the majority of recovered sequences are of unknown function, inferences requiring comparisons of the relative frequency of genes involved in responses to biotic and abiotic stresses are speculative.

**Figure 3:**
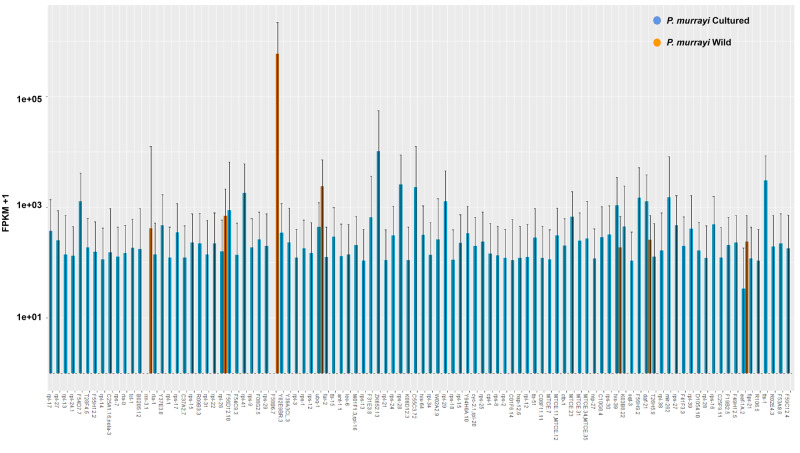
Comparison of gene expression between lab-reared and wild-caught populations of *P. murrayi* (*P* < 0.05), top 100 statistically most significant differentially expressed genes (*P* < 0.05).

We obtained an original RNAseq data size of 4.3 Gigabytes (G) for cultured *P. murrayi* and 1.94 G for wild-caught *P. murrayi*. We predicted functional genes of *P. murrayi* using both *ab initio* annotation without a reference, and annotation with *C. elegans* as a reference. A comparison of wild-caught and cultured *P. murrayi* transcriptomes revealed that the cultured population has more up-regulated genes compared to the wild-caught ones, especially for genes related to growth and development, while genes related to freeze tolerance and cellular metabolism are down-regulated in cultured *P. murrayi* (Figure 3). Among the top 100 significantly differentially expressed genes, only 5 occur in the wild-caught field samples, whereas the vast majority of up-regulated genes are from the lab-reared population.

The gene *rla-1*, which is highly expressed in wild-caught *P. murrayi* relative to the cultured populations, plays a role in ribosome synthesis in animals. The genes *far-2* and *Y82E9BR.3* exhibit elevated expression levels in wild-caught *P. murrayi*. These genes, which have orthologues in *C. elegans*, *C. nigoni* and *Toxocara canis*, are related to ATP synthesis (Table 7).

**Table 7:** Genes up-regulated in wild-caught *P. murrayi* among top 100

Gene Name	Function
*rla-1*	Structural constituent of ribosome (Hu *et al.* 2019)
*far-2*	Fatty acid and retinol binding (Hu *et al.* 2019)
*fipr-21*	Unknown function
*T26H5.9*	Unknown function
*Y82E9BR.3*	Mitochondrial membrane ATP synthase (Gaudet *et al.* 2011; Xu *et al.* 2018)

## Discussion

Based on the most complete phylogenomic analysis of the Nematoda to date (Blaxter and Koutsovoulos 2015; Koutsovoulos 2015), *P. murrayi* is the sister taxon to the Secernentea, and as such plays a key role in determining character polarity and understanding the evolutionary radiation of the Secernentea. The Secernentea are the evolutionary lineage from which *C. elegans* and virtually all of the major plant and animal parasitic clades of nematodes arose, many of which are scientifically and economically important, and/or serve as model species for molecular, developmental and genetic studies (Gao *et al.* 2008; Adhikari *et al.* 2009; Coolon *et al.* 2009; Kim *et al.* 2014). Aside from the fact that *C. elegans* is a model nematode, it is also a free-living, bactivorous soil nematode, which makes it ecologically comparable to *P. murrayi*. As the best-studied metazoan genome, *C. elegans* provides an optimal reference for predicting *P. murrayi* gene functions.

*Plectus murrayi* tolerates a number of environmental stresses which are much harsher than those encountered by most free living nematodes (Adams *et al.* 2014a), making it an ideal model for understanding the evolution of survival strategies under multiple and strong environmental stresses. Meaningful inferences about gene and genome evolution in *P. murrayi* will require comparisons of more closely related taxa using phylogenetic comparative methods (Brooks and McLennan 1991; Harvey and Pagel 1991), but we speculate that many of the observed differences are evolutionary responses driven by their austere environment.

Genome decay is a known evolutionary response to environmental stress (Marguerat *et al.* 2014; Faddeeva-Vakhrusheva *et al.* 2016), and increased use of alternative splicing could be a way for organisms to generate additional gene products and mitigate the loss of functional genes (Mandadi and Scholthof 2015; Ma *et al.* 2018). We found that *P. murrayi* has fewer genes than *C. elegans* and the intron retention percentage is higher in *P. murrayi* than in *C. elegans* (Table 4), which suggests that one way to respond to stress could be by shifting alternative splicing strategies. To increase energy efficiency, *P. murrayi* may have reduced its number of expressed genes, instead using introns more efficiently for coding functional genes in an environment with limited elemental resources (Schmitz *et al.* 2017; Filichkin *et al.* 2018). Some studies have shown that intron retention occurs commonly in plants exposed to environmental stress (cold or heat) (Filichkin *et al.* 2018). In the case of *P. murrayi*, intron retention may have been coopted to increase the expression of genes required for stress survival, including the high number of genes involved in biogenesis and metabolic processes (Peter *et al.* 2011; Pang *et al.* 2017; Kalinina *et al.* 2018; Palud *et al.* 2018). Thus, intron retention may be an evolved strategy to enable *P. murrayi* to generate different transcripts from fewer genes, getting around the problem of genome decay.

Researchers have suggested that variation in organismal stoichiometry is related to the availability of key nutrients (*e.g.* carbon, nitrogen and phosphorus; CNP) in the environment (Elser *et al.* 2000; Sterner and George 2000; Zimmerman *et al.* 2014). Mechanisms underlying the observed variation in biological stoichiometry can be coordinated by a combination of genetic and environmental drivers (Carroll *et al.*; Mathis et al. 2017). Environmental CNP stoichiometry influences the expression of genes related to nematode growth and development, affecting trophic interactions ultimately resulting in subsequent feedbacks on nutrient cycling in the environment (Elser *et al.* 1996, 2000; Sterner and George 2000). Antarctic soil ecosystems exhibit CNP ratios that can be severely skewed and limiting (Barrett *et al.* 2007). Thus, soil organisms that live in these ecosystems might lower the expression of some genes associated with growth and development in order to use available CNP more efficiently (Elser *et al.* 1996).

In the transcriptomic comparison of lab-reared and wild-caught *P. murrayi*, we found that most of the genes related to metabolism and cellular processes, such as *tts-1*, *daf-2*, *sqt-3*, *his-39*, *ttr-51*, *lec-6*, *ant-1*, *far-2*, are up-regulated in the lab-reared *P. murrayi* compared to stoichiometrically constrained animals that have not undergone generations of artificial selection under laboratory conditions (Figure 3). This suggests that lab-reared populations may be adjusting gene expression as an evolutionary response to the ameliorated environmental conditions in the laboratory. Further, we found that genes associated with growth and development, such as ATP synthesis, ribosome synthesis, and lipid binding were up-regulated in wild-caught *P. murrayi*. This pattern suggests that the wild-caught populations are more efficient at using the nutrients and energy that are more limited in their natural environment.

The draft genome of *P. murrayi* provides an important baseline for understanding the evolutionary origin and maintenance of traits that have allowed it to persist in one of the most extreme soil environments on the Earth. The harsh, nutrient-limited soils of continental Antarctica have likely shaped the evolutionary histories of its fauna in profound but predictable ways, and it is not unreasonable to expect these influences to be reflected in their genomic architecture. As a baseline for future work involving comparisons among other Antarctic taxa, we hope that the draft genome of *P. murrayi* can be used to test hypotheses of evolutionary genomic trends. Such an understanding will be important not only for revealing the molecular genetic processes that are consistent with historical and contemporary patterns of genome evolution, but also for predicting how these organisms may respond to the dramatic environmental changes that are predicted to occur with amplified climate change over the decades to come.
